# Signal-dependent fra-2 regulation in skeletal muscle reserve and satellite cells

**DOI:** 10.1038/cddis.2013.221

**Published:** 2013-06-27

**Authors:** N S Alli, E C Yang, T Miyake, A Aziz, H Collins-Hooper, K Patel, J C McDermott

**Affiliations:** 1 Department of Biology, York University, 4700 Keele Street, Toronto, Ontario, Canada; 2 Muscle Health Research Centre (MHRC), York University, 4700 Keele Street, Toronto, Ontario, Canada; 3 Centre for Research in Biomolecular Interactions (CRBI), 4700 Keele Street, Toronto, Ontario, Canada; 4 Sunnybrook Health Sciences Centre, 2075 Bayview Avenue, Toronto, Ontario, Canada; 5 School of Biological Sciences, Hopkins Building, University of Reading, Whiteknights Campus, Reading, Berkshire, UK; 6 Centre for Research in Mass Spectrometry (CRMS), York University, 4700 Keele Street, Toronto, Ontario, Canada

**Keywords:** activator protein-1, phosphorylation, myogenesis, satellite cells, ERK 1/2 signaling

## Abstract

Activator protein-1 (AP-1) is a ubiquitous transcription factor that paradoxically also has some tissue-specific functions. In skeletal muscle cells, we document that the AP-1 subunit, Fra-2, is expressed in the resident stem cells (Pax7-positive satellite cells) and also in the analogous undifferentiated ‘reserve' cell population in myogenic cultures, but not in differentiated myofiber nuclei. Silencing of Fra-2 expression enhances the expression of differentiation markers such as muscle creatine kinase and myosin heavy chain, indicating a possible role of Fra-2 in undifferentiated myogenic progenitor cells. We observed that Fra-2 is a target of cytokine-mediated extracellular signal-regulated kinase-1/2 signaling in cultured muscle cells, and extensive mass spectrometry and mutational analysis identified S320 and T322 as regulators of Fra-2 protein stability. Interestingly, Fra-2 S320 phosphorylation occurs transiently in activated satellite cells and is extinguished in myogenin-positive differentiating cells. Thus, cytokine-mediated Fra-2 expression and stabilization is linked to regulation of myogenic progenitor cells having implications for the molecular regulation of adult muscle stem cells and skeletal muscle regeneration.

Skeletal muscle development (myogenesis) is a complex and crucial process in all metazoan embryonic development involving temporal and spatial coordination of a network of myogenic transcription factors.^1^ In particular, members of the myogenic regulatory factor family have a prominent role during myogenesis. These transcription factors include commitment factors MyoD and Myf5, and differentiation markers myogenic regulatory factor4 and myogenin.^2, 3, 4, 5^ Other transcription factors, such as members of the myocyte enhancer factor 2.^6, 9^ Six and Smad families, also have obligatory roles in regulating skeletal myogenesis.^10, 11^ Along with the tissue-restricted myogenic factors, contributions of more ubiquitous transcriptional regulators at muscle promoters also impinge on the precise combinatorial regulation required for orchestration of the myogenic program.^12^ Previously, we have implicated activator protein-1 (AP-1) as one example of this class of ubiquitous transcription factors that contribute, in concert with the myogenic regulatory factors, to the regulation of muscle gene expression.

AP-1 is a transcription factor complex comprising of Jun (c-Jun, JunD and JunB) homodimers or Jun-Fos (Fra-1, Fra-2, c-fos and FosB) heterodimers. AP-1 complexes have been classically associated with cancer progression and are characterized as proto-oncogenes.^13, 14^ However, they are ubiquitously expressed and have a wider role than initially anticipated in the control of tissue-specific genes. In myogenic cells, previous data has suggested that AP-1 is a negative regulator of differentiation based on observations that c-Jun dimerizes with MyoD to inhibit its activity,^15^ and also MyoD is required to downregulate c-Fos for differentiation to proceed.^16^ The AP-1 subunits are well-defined nuclear targets of signaling pathways active in skeletal muscle, such as mitogen-activated protein kinases (MAPK), and extracellular signal-regulated kinase (ERK) 1/2 is a known upstream kinase of Fos family members.^17, 18^

The AP-1 complexes that bind DNA in skeletal muscle cells was previously examined by us and it was found that Fra-2 was a primary component of the myogenic AP-1 DNA binding complex.^12^ Thus, we sought to assess the role of Fra-2 and determine the mechanism by which it is regulated during myogenesis. Cardiotrophin-1 (CT-1), is a cytokine that potently inhibits skeletal muscle differentiation by activating MEK 1/2–ERK 1/2 signaling.^11^ Using a mass spectrometry-based approach along with mutational analysis, we report two regulatory ERK 1/2 MAPK phosphorylation sites on Fra-2 in response to CT-1–ERK signaling. The two Fra-2 phosphorylation sites, S320 and T322, contribute to a potentiation in Fra-2 protein stabilization. These sites are phosphorylated in response to growth factors such as CT-1. Stabilization of Fra-2 by phosphorylation results in an overall inhibition of differentiation, whereas loss-of-function studies using Fra-2 siRNAs demonstrated that reduced Fra-2 levels potentiate expression of muscle-specific marker genes such as muscle creatine kinase and myosin heavy chain (MyHC). Interestingly, separation of reserve cells and myotubes (MT) in a differentiated culture revealed differential expression of Fra-2. Fra-2 expression is largely restricted to reserve cells during differentiation, and further studies indicate that Fra-2 is expressed and phosphorylated in activated muscle satellite cells, the adult muscle stem cell progenitor population.

## Results

### Fra-2 is a downstream target of cytokine-activated cellular signaling pathways that inhibit myogenic differentiation

**Figure 1:**
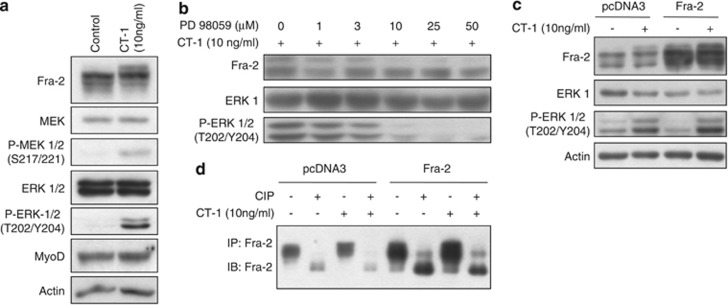
Fra-2 is a downstream target of the MEK 1/2–ERK 1/2 MAPK pathway. (**a**) C2C12 MB were serum starved for 8 h before treatment with CT-1 (10 ng/ml) or solvent control. Cells were harvested after 20 min of CT-1 treatment and Fra-2 expression was assessed by western blotting. (**b**) Proliferating MB following serum withdrawal for 8 h were pre-treated with PD 98059 1 h before CT-1 (10 ng/ml) treatment. Cells were harvested 20 min after CT-1 treatment and analyzed for Fra-2 expression by western blotting. (**c**) Western blot of exogenously expressed Fra-2 in cells treated with CT-1 (10 ng/ml). C2C12 MB-expressing exogenous Fra-2 were incubated with CT-1 (10 ng/ml) for 20 min before harvesting. Total cell lysates were prepared and Fra-2 was IP from cells. (**d**) The IP samples were treated with CIP or its buffer before western blotting

Expression of the AP-1 transcription factors has been observed in differentiating myogenic (C2C12) cells even though exogenous expression of c-Jun has been shown to repress myogenesis.^19^ We previously determined that Fra-2 is the main Fos-related subunit of the AP-1 DNA binding complex in myogenic cells and its regulation may be mediated by phosphorylation.^12^ Fra-2 is known to be a ERK 1/2 MAPK target,^20, 21^ and the ERK 1/2 MAPK pathway is active in proliferating myoblast (MB) cells and in later stages of differentiation, where it is critical for MB fusion.^22^ Growth factors that suppress differentiation are involved with the maintenance of the undifferentiated state such as CT-1.^11^ These growth factors, specifically CT-1, have also been shown to activate ERK 1/2, and we therefore postulated that Fra-2 may be a downstream target in myogenic cells. When MB in serum-reduced conditions were treated with CT-1 (10 ng/ml), we observed an increase in P-ERK 1/2 10–30 min post treatment (see Supplementary figure S1). Concurrently, we observed a low-mobility Fra-2 band that was correlated with the activation of ERK 1/2 in CT-1-treated cells (see Supplementary figure S1). Our data indicate that CT-1 was able to activate the MEK 1/2–ERK 1/2 MAPK pathway and target Fra-2 by inducing a slower migrating Fra-2 band in C2C12 cells (Figure 1a). To test whether the lower mobility Fra-2 band was ERK 1/2 dependent, we used an inhibitor of activated MEK 1/2 (PD 98059) to block ERK 1/2 activation. Figure 1b indicates that the slower mobility Fra-2 band seen with CT-1 (10 ng/ml) treatment was reduced by the addition of the MEK 1/2 inhibitor PD 98059 and was correlated with reduced P-ERK 1/2 levels. Thus, repression of ERK 1/2 activation by pharmacological inhibition of MEK 1/2 resulted in a loss of the modified form of Fra-2 in C2C12 cells. These data implicate ERK 1/2 activation by CT-1 with changes in the mobility of Fra-2 and repression of myogenesis.

To directly test whether the change in Fra-2 mobility with CT-1 was due to direct phosphorylation, we analyzed mobility changes of endogenous and exogenously expressed Fra-2 in the presence or absence of CT-1 in C2C12 MB cells (Figure 1c). Fra-2 was immunoprecipitated from MB and treated with calf intestinal phosphatase (CIP), which resulted in the loss of the slower mobility Fra-2 band (Figure 1d). Interestingly, in addition to loss of the higher mobility Fra-2 band, we observed enhanced mobility of all the immunoreactive Fra-2 after CIP treatment, suggesting that Fra-2 exists in various states of phosphorylation (Figure 1d).

### Identification and characterization of ERK 1/2-specific Fra-2 phosphorylation sites

**Figure 2:**
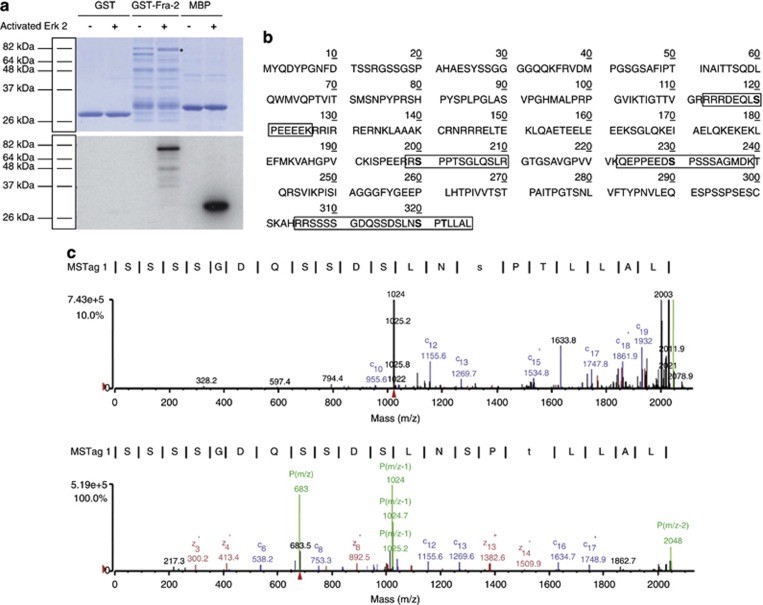
Identification of Fra-2 phospho-acceptor sites using phospho-peptide mass spectrometry analysis. (**a**) Purified GST, GST-Fra-2 or MBP were incubated with *γ*-^32^P-ATP and activated ERK 2 or its buffer *in vitro*. The samples were run on an 8% SDS-PAGE, which was Coomassie stained (**a**), dried and exposed to film (**b**). GST alone was used as a negative control and MBP was used as a positive control. (**b**) Linear Fra-2 amino acid sequence. The *in vitro* kinase assay was repeated with GST-Fra-2, activated ERK 2 and unlabeled ATP. Samples were resolved on an 8% SDS-PAGE, and the unphosphorylated and phosphorylated GST-Fra-2 bands were excised, digested by trypsin and the resultant peptides were analyzed by mass spectrometry. The results from the mass spectrometry analysis are summarized: where phosphopeptides detected by mass spectrometry are boxed, DEF domain (282-285) is underlined and ERK 2 phosphorylated residues (120, 200, 230, 320, 322) are in bold. (**c**) Spectra for S320 (upper panel). C13 ion has a m/z of 1269.7 and C15 ion has a m/z of 1534.8, and a difference of 265.1. The C13 ion is composed of S and P that have masses of 87.08 and 97.12, respectively, added together to give a value of 184.2. The difference between 265.1 and 184.2 is 80.90, which corresponds to a addition of a phosphate group. The spectra for T322 (lower panel) has a C13 of 1269.6 and C16 of 1634.7, and has a difference of 365.1. The C16 ion corresponds to S, P and T, which m/z of 87.08, 97.12, and 101.11, respectively, added together gives 285.31. The difference of the value gives 79.79, corresponding to an addition of a phosphate group

As there is always some ambiguity as to the true identity of kinase–substrate interactions in cell lysates, we performed an *in vitro* kinase assay to assess if Fra-2 was directly phosphorylated by activated ERK 1/2. Figure 2a (upper panel) shows that when glutathione S-transferase (GST)-Fra-2 was incubated with activated ERK 2 and *γ*-^32^P-ATP, a change in mobility of the GST-Fra-2 protein was seen in the Coomassie blue-stained gel (as marked by asterisks in the upper panel) in agreement with the cell-based studies. In addition, the slower mobility complex was hyperphosphorylated as indicated by *γ*-^32^P-ATP radioactive incorporation into the Fra-2 protein when the gel was exposed by autoradiography (Figure 2a lower panel), thus, directly showing that Fra-2 was phosphorylated by ERK 2.

To define the effects of ERK 1/2 on Fra-2, we mapped the specific phosphorylation sites on Fra-2 by ERK 2 using mass spectrometric methods. We performed an *in vitro* kinase assay using GST-Fra-2 as substrate and ERK 2 as kinase, and processed the Coomassie blue-stained Fra-2 protein bands for mass spectrometric analysis. Using this methodology, four phosphorylated peptides were detected (boxed in Figure 2b) containing four proline-directed phosphorylated residues: S120, S200, S230 and S320, and one potential non-proline-directed site T322 (bold in Figure 2b) were identified. These phosphopeptides were selected and fragmented by tandem MS. Figure 2c (upper panel) shows an ETD MS/MS spectrum of a precursor peptide (m/z 1023.5 doubly charged), which corresponds to amino acids 317-326 of Fra-2 with an extra 80 Da. Note that in the ETD spectrum, fragments from the N terminus give C ion series and fragments from the C terminus give Z ion series, and the peptide bond N-terminal to a proline residue does not break. The observed C ion series (C10, C12, C13, C15, C17, C18 and C19) supported the identity of the peptide (317- SSSSGDQSSDSLNSPTLLAL-326). In addition, though fragment C14 was not expected because of the nature of a proline residue in the ETD spectrum, the difference between the C13 (1269.7 m/z) and C15 (1534.8 m/z) fragment ions is 265.1 Da, which is equal to a combination of theoretical mass of serine and proline, and an extra 80 Da. Thus, it is clear that S320 was phosphorylated in this ETD spectrum. Similarly in Figure 2c (lower panel), the C13 ion had a m/z of 1269.6 Da and C16 of 1634.7 Da. The difference between the two ion fragments is 365.1 Da, which is the mass of serine, threonine and proline. The theoretical mass of serine, threonine and proline is 285.31 Da, and as the difference between the observed (365.1 Da) and the actual (285.31 Da) fragment mass was 80 Da, we can conclude that the serine or threonine is phoshorylated. Thus, these data support phosphorylation at S320 and T322 on Fra-2.

### Mutational analysis of Fra-2-specific phosphorylation sites

**Figure 3:**
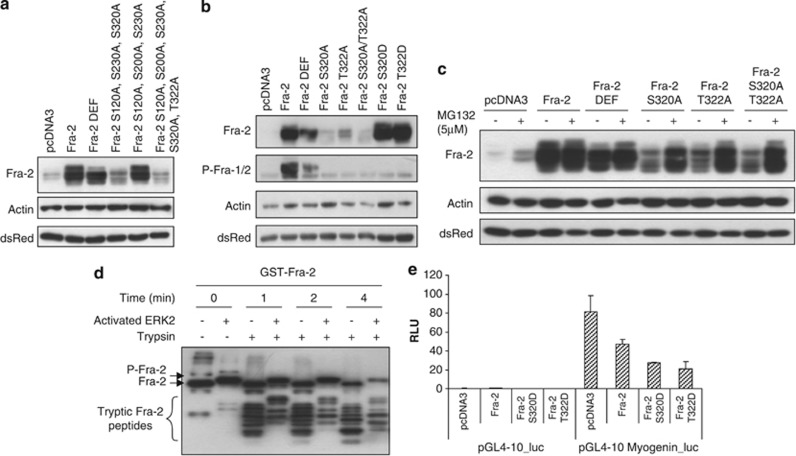
Expression and stability of Fra-2 phospho-mutants in myogenic cells. (**a**) Western blot analysis showing the expression of Fra-2 (wild-type), Fra-2 DEF and mutated proteins: Fra-2 S120A, S230A, S320A, Fra-2 S120A, S200A, S230A, Fra-2 S120A, S200A, S230A, S320A and T322A. (**b**) Western blot analysis showing expression of Fra-2 (wild-type), Fra-2 DEF, Fra-2 S320A, Fra-2 T322A, Fra-2 S320A/T322A, Fra-2 S320D and Fra-2 T322D in myogenic cells. (**c**) C2C12 cells treated with MG132 (5 *μ*M) for 5 h. Cells were harvested and expression of wild-type and mutated Fra-2 were analyzed by western blotting. Actin was used as a loading control and dsRed was used as a marking of transfection efficiency. (**d**) Limited proteolytic digestion of GST-Fra-2. An *in vitro* kinase assay using 10 *μ*g of GST-Fra-2 and 20 ng of activated ERK 2 was performed. The reaction was divided into seven tubes and trypsin was added at a ratio of 100 : 1 and incubated for the indicated times. Samples were run on a 10% SDS-PAGE and Fra-2 was detected by western blotting. (**e**) C2C12 cells were transfected with wild-type Fra-2, Fra-2 S320D or T322D, and a myogenin promoter reporter gene (pGL4-10-*myogenin*-luc). Cells were maintained in DM conditions for 24 h before harvesting. Luciferase values were normalized to renilla

We next sought to determine the function of the Fra-2 phosphorylation sites identified by mass spectrometry. To address this, a series of combinatorial Fra-2 phospho-acceptor site mutations were generated by site-directed mutagenesis. Some interesting observations were made when the combinatorial mutations were expressed in C2C12 cells. Fra-2 phospho-mutations containing S320A and/or the T322A mutation altered the Fra-2 protein mobility pattern compared with wild-type Fra-2, which was similar to Fra-2 DEF, which contains a mutation in the ERK 1/2 docking site. Fra-2 containing S320A and/or T322A had an observable loss of the slower migrating hyperphosphorylated Fra-2 band, whereas mutations not containing S320A or T322A did not (see Supplementary figure S2). Interestingly, expression levels of Fra-2 containing S320A and/or T322A mutations were expressed at lower levels in addition to the loss of the slower mobility (High-Mr) band (Figure 3a). Initial mutational data suggested that S320 and T322 might be important phosphorylation sites for Fra-2 stability. Interestingly, S320 is a conserved phosphorylation site on Fra-1 and a similar observation was previously reported;^23^ however, the T322 site has not been examined in any Fos family member. In order to assess the function of these sites, single neutralizing mutation of S320 and T322 to alanine were generated and expressed in C2C12 cells. Neutralization of S320 or T322 alone resulted in loss of the slower mobility band and reduced notably expression of Fra-2 (Figure 3b). The double mutation, both S320A and T322A, had similar effects to the single mutations. These results suggest that S320 and T322 are responsible for hyperphosphorylation of Fra-2; moreover, lack of phosphorylation at these sites contributes to a marked decrease in Fra-2 protein levels compared with wild-type Fra-2. Interestingly, we noticed that neutralization of S320 and T322 to alanine causes Fra-2 to become considerably less stable than wild-type Fra-2 (Figure 3b), and we therefore postulated that phospho-mimetic mutation would result in the opposite effect. Expression of S320D and T322D mutants reconstituted Fra-2 stability similar to wild-type Fra-2 (Figure 3b), suggesting that phosphorylation at S320 and T322 is required for Fra-2 protein stability. To confirm phosphorylation of Fra-2 on S320, we used a phospho-Fra-1 antibody that reacts with the conserved peptide containing phospho-S320 of Fra-2. Wild-type Fra-2 when phosphorylated at S320 could be detected at high levels using the phospho-specific antibody, indicating its efficacy (Figure 3b). As expected, Fra-2 S320D was undetectable with the phospho-specific antibody, but unexpectedly, cells expressing Fra-2 T322D did not have a high level of phosphorylated S320 (Figure 3b). Moreover, we observed a reduction in phospho-S320 in Fra-2 when only T322 was mutated, suggesting that T322 may be required for efficient phosphorylation of S320.

### Fra-2 phosphorylation at S320 and T322 regulates protein stability

As our earlier experiments suggested that Fra-2 phosphorylation might alter its function in myogenic cells, the Fra-2 phospho-mutations were tested in MBs. We expressed the phospho-mutations S320A and T322A in MB and MT, and observed reduced expression of the mutated versions compared with wild-type Fra-2 in MB and 72 h MT (see Supplementary figure S3), suggesting that S320 and/or T322 are important sites for stabilizing Fra-2 throughout differentiation. Loss of phosphorylation at S320 and T322 destabilized Fra-2, possibly targeting it to the proteosome for degradation. To address this further, we treated C2C12 expressing the mutated Fra-2 variants with the proteosome inhibitor MG132. When MG132 was included, we noticed that the expression of the phospho-neutralizing mutations increased, as well as wild-type Fra-2 and Fra-2 DEF (Figure 3c). Thus, Fra-2-targeted proteosomal degradation is resisted by S320 and T322 phosphorylation. Our data suggest that CT-1 can activate the ERK 1/2 MAPK pathway, which phosphorylates Fra-2 at S320 and T322 affecting protein stability.

Phosphorylation can result in conformational changes in protein structure with, in many cases, subsequent effects on function. Fra-2 protein is stabilized when phosphorylated by ERK 1/2, and we postulated that its conformation in the phosphorylated and unphosphorylated states may differ. When unphosphorylated at S320 and T322, Fra-2 is unstable. To determine if the phosphorylated form of Fra-2 is in a different conformation to the unphosphorylated protein, we performed a limited proteolytic digestion assay. The idea of this assay is that if a protein is in an altered conformational state, then its susceptibility to limited proteolytic digestion may be altered. After incubating GST-Fra-2 with activated ERK 2 kinase, we observe a phosphorylated high-mobility Fra-2 band (Figure 3d). When digested for short time periods with trypsin, we observe different patterns of peptide accumulation for the unphosphorylated and phosphorylated forms of GST-Fra-2 indicative of a change in conformation (Figure 3d). This analysis provides initial structural evidence that ERK 1/2 phosphorylated Fra-2 is in an altered conformation compared with the unphosphorylated Fra-2. Further NMR and crystallography-based studies will be required to fully characterize the ERK 1/2-dependent conformational changes in Fra-2.

Stability of Fra-2 is potently enhanced by phosphorylation at S320 and T322, and the next question concerns how this affects its function. Therefore, we investigated whether stability affected Fra-2 function during myogenesis. The myogenin promoter is active in differentiation conditions and its activity is regulated by MyoD. Using myogenin as a marker for skeletal muscle differentiation, we found that wild-type Fra-2 inhibited the induction of the myogenin promoter, and Fra-2 S320D and T322D mutations further blocked this activity (Figure 3e) compared with the control. Thus, suggesting that stability of Fra-2 by phosphorylation may impede myogenesis.

### Loss of Fra-2 expression enhances myogenesis

**Figure 4:**
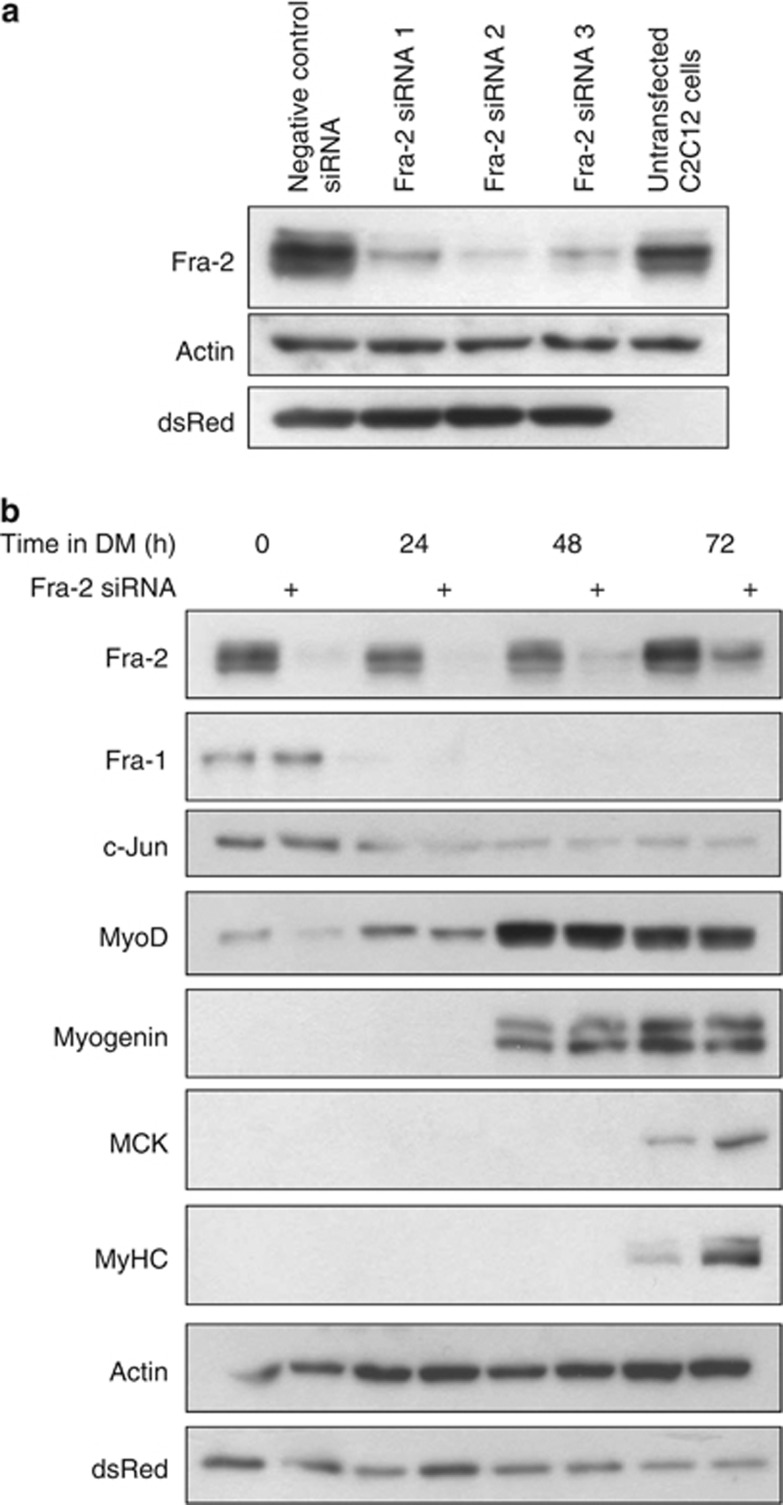
Knockdown of Fra-2 enhances differentiation. (**a**) Three independent siRNAs targeting Fra-2 were transfected in proliferating C2C12 cells. Western blot analysis was performed to determine level of Fra-2 knockdown. Actin was used as a loading control and dsRed as a marker for transfection efficiency. (**b**) Knockdown of Fra-2 was assessed during a time course of C2C12 differentiation by western blotting. Protein levels of some AP-1 components and myogenic markers were also analyzed

In an initial attempt to assess the functional role for Fra-2 in skeletal myogenesis, we silenced its expression using siRNA technology. Initially, three siRNAs targeting Fra-2 were characterized for their efficacy. All three siRNAs reduced Fra-2 protein level when compared with cells transfected with a negative control siRNA and untransfected cells; however, siRNA2 gave the most optimal reduction (Figure 4a). Fra-2 suppression in differentiating MB cultures resulted in no change in Fra-1 or c-Jun expression (Figure 4b) during a time course of skeletal muscle differentiation. In conditions where Fra-2 was reduced, we observed an increase in late differentiation marker proteins muscle creatine kinase and MyHC compared with control conditions (Figure 4b). Thus, a reduction of Fra-2 protein levels resulted in an observable increase in the expression of muscle-specific differentiation markers.

### Fra-2 expression in reserve and primary satellite cells

**Figure 5:**
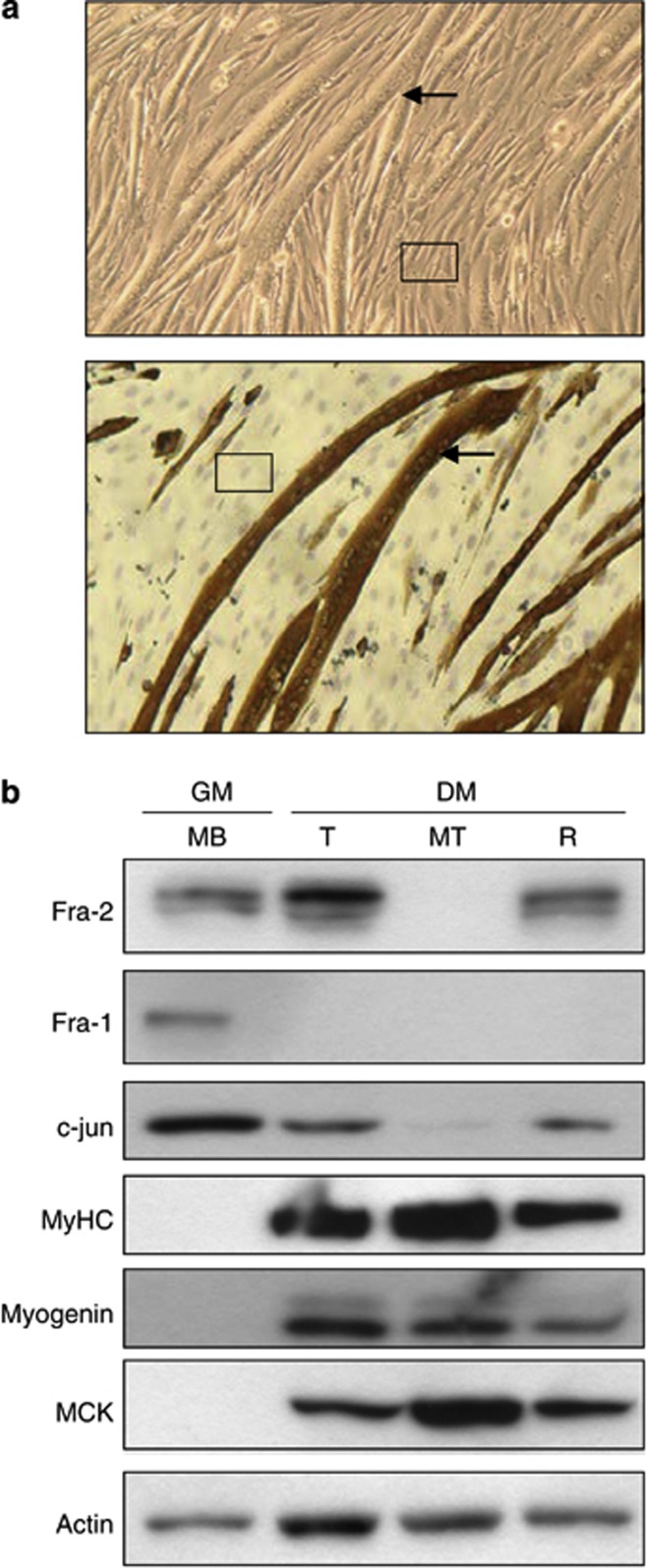
Expression and localization of Fra-2 in myogenic cells. (**a**) A 96 h culture of differentiated C2C12 cells under phase contrast microscopy. Upper panel depicts cells in culture maintained in DM and the lower panel shows fixed cells immunostained for MyHC, a marker of differentiation, the nuclei were stained with hemotoxylin. (**b**) Western blot analysis was performed on proliferating C2C12 cells in growth media (GM) conditions and differentiated cultures maintained in DM. The differentiated cultures were analyzed by total (T), which included all cells in a differentiated culture, MT, the fraction enriched for MT, and the reserve (R) fraction of mononucleated cells

In a differentiated culture of C2C12 cells as shown in Figure 5a, there is a bimodal population of cells: multinucleated MTs (indicated by an arrow) and mononucleated ‘reserve cells' (as seen boxed). The establishment of the reserve population is a stochastic event, as this is a clonal cell line in which all cells have the same potential when seeded, but yet some form MT, whereas others remain quiescent and undifferentiated. Reserve cells and MT in a differentiated culture can be separated to assess differential gene expression.^24, 25, 26^ When separated, the reserve cells and MT revealed differential expression of AP-1. Protein analysis in Figure 5b shows that Fra-2 was expressed in proliferating MB in growth media and in total differentiated lysate (includes MT and reserve cells). However, when MT and reserve cells were fractionated from a differentiated culture and western blotting was performed, we noticed that Fra-2 (along with c-Jun) was greatly reduced in MT, whereas their expression was maintained in the reserve cells (Figure 5b). Interestingly, Fra-1 expression was detected in only MB cells, ruling out its involvement during differentiation. As Fra-2 is not expressed in MT, we attributed potentiation of differentiation to a reduction of the reserve cell population, suggesting that Fra-2 ordinarily holds the cells in an undifferentiated state and, when its expression is extinguished, leads these cells to differentiate.

**Figure 6:**
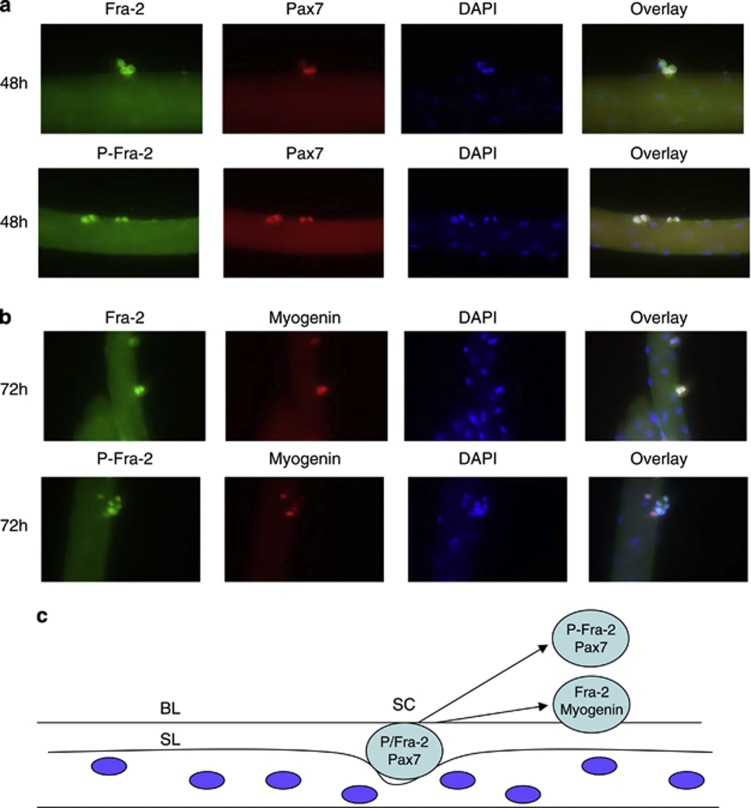
Fra-2 expression in satellite cells. Myofibers were dissected from EDL muscle of adult mice and cultured in a dish for the indicated times (48 and 72 h). (**a**) Fibers were immunostained for Fra-2 (green) or P-Fra-2 (green), and Pax7 (red) at 48 h in culture. Nuclei were stained with DAPI (blue). (**b**) Fibers were immunostained for Fra-2 (green) or P-Fra-2 (green), and myogenin (red) at 72 h in culture. Nuclei were stained with DAPI (blue). (**c**) Schematic representation of Fra-2, P-Fra-2, Pax7 and myogenin expression in satellite cells (SC) in single-fiber cultures. Dark blue circles represent myonuclei and light blue satellite cells that reside within the basal lamina (BL) and sarcolemma (SL)

Reserve cells have been described as analogous to satellite cells, which lie within the basal lamina of muscle fibers and are activated after muscle injury. We performed immunostaining for Fra-2 and P-Fra-2 on primary muscle fibers at various time points and stained for Pax7 and myogenin (Figures 6a, b). In view of the analogy between reserve cells and satellite cells, we postulated that Fra-2 might have a role in satellite cells. To address this, we used primary single-fiber cultures in which myofibers are cultured with their associated satellite cells. In these cultures, satellite cell activation and differentiation occurs in a manner similar to their activation *in vivo*. Fra-2 and its phosphorylated form were expressed at 48 h in Pax7-positive cells and was not expressed in the myonuclei of the myofiber consistent with our idea that Fra-2, along with Pax7, marks satellite cells (Figure 6a). At 72 h, myogenin expression was observed in cells that co-expressed Fra-2 (Figure 6b). Interestingly, the Myogenin expressing cells did not co-express the phosphorylated form of Fra-2 (Figure 6b). These data indicated that Fra-2 is expressed in both Pax7 and myogenin-positive satellite cells, whereas phospho-Fra-2 was only observed in cells expressing Pax7 but not myogenin (Figure 6c). Together, these data suggest that Fra-2 phosphorylation only marks activated satellite cells, and the phosphorylation is extinguished in myogenin-positive differentiating cells.

### ERK 1/2 inhibition modulates satellite cell differentiation and Fra-2 phosphorylation

**Figure 7:**
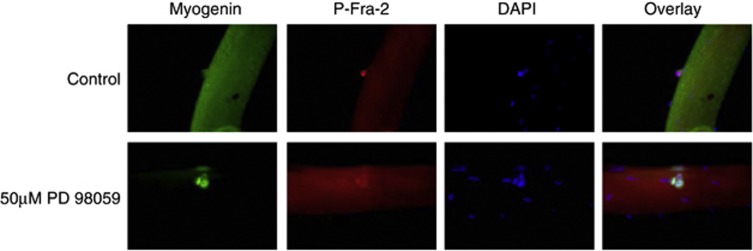
MEK 1/2 inhibition activates satellite cell differentiation in primary muscle fibers. Satellite cells in primary single muscle fibers from the EDL muscle were isolated from adult mice. After 48 h in culture, fibers that were previously incubated with 50 *μ*M of the MEK 1/2 inhibitor PD 98059 or its control (DMSO) were immunostained for myogenin (green) or P-Fra-2 (red). DAPI (blue) was used to mark nuclei

**Figure 8:**
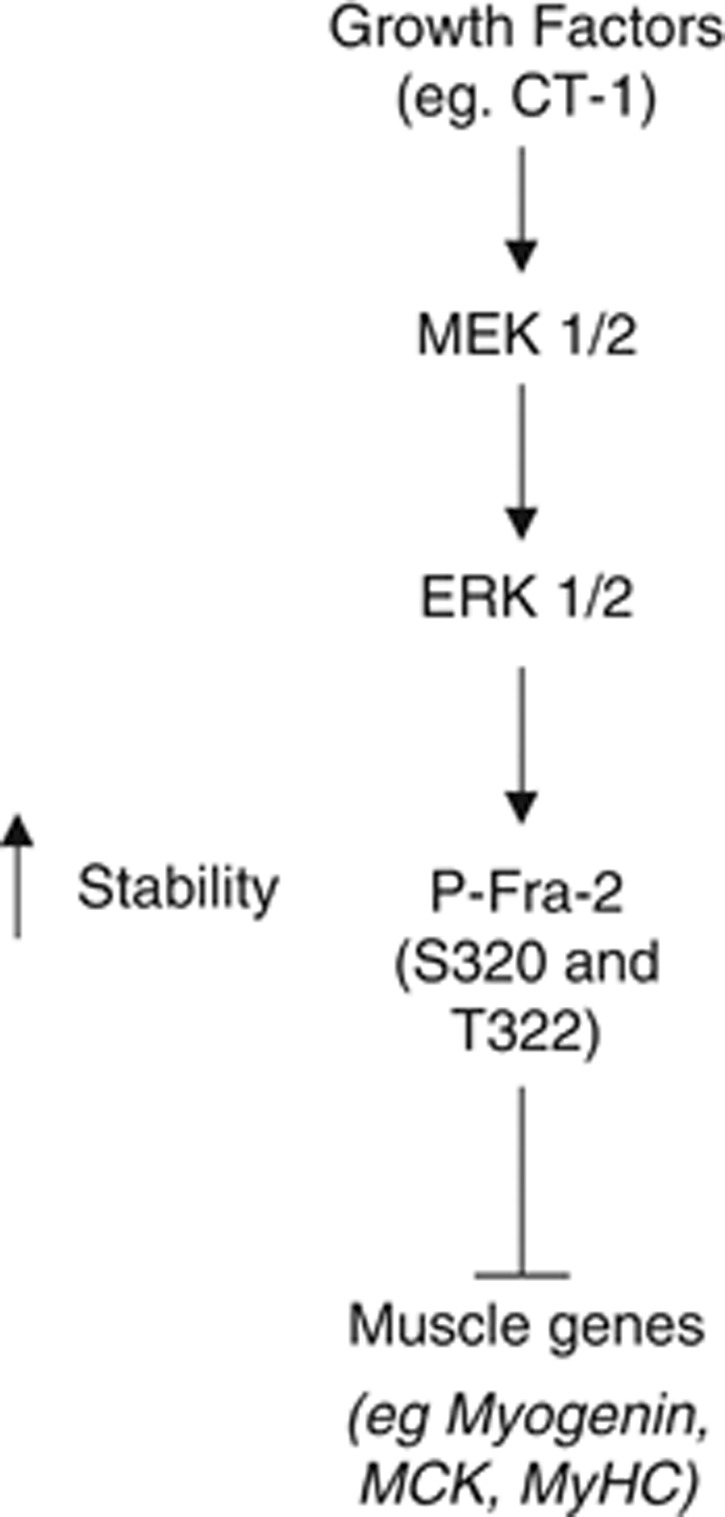
Fra-2 phosphorylation may regulate muscle gene induction in C2C12 cells. Fra-2 is phosphorylated at S320 and T322 by ERK 1/2, resulting in protein stabilization, which leads to inhibition of muscle-specific gene expression and differentiation

In 48 h, primary single-fiber cultures P-Fra-2 is expressed in Pax7-positive satellite cells, but myogenin expression does not appear until 72 h in culture and is not co-expressed with P-Fra-2. When 50 *μ*M of the MEK 1/2 inhibitor, PD 98059, was added to 48 h primary culture fibers, P-Fra-2 levels decreased due to ERK 1/2 activation being inhibited (Figure 7). In addition, myogenin expression appeared precociously at 48 h in fibers treated with PD 98059 compared with control-treated fibers (Figure 7). Taken together, these experiments are consistent with the idea that ERK phosphorylation and Fra-2 stabilization are congruous with the undifferentiated state in MBsand satellite cells. Conversely, repression of ERK signaling and concomitant hypophosphorylation and destabilization of Fra-2 result in enhanced myogenic differentiation (Figure 8). The identification of Fra-2 expression in both ‘reserve' and satellite cells may have important implications for a role in muscle maintenance and regeneration.

## Discussion

In this study, we report a novel regulation of the AP-1 subunit Fra-2 in skeletal myogenesis. We have demonstrated that Fra-2's expression is restricted to mononucleated ‘reserve cells' in a differentiated culture, suggesting a possible role in maintaining the undifferentiated state. In addition, we have identified an important level of regulation for AP-1 complex composition by identifying S320 and T322 as ERK 1/2-dependent phosphorylation sites on Fra-2 in skeletal muscle. These phospho-acceptor sites determine the contribution of Fra-2 to the AP-1 complex by potently stabilizing Fra-2 protein levels. Neutralization of one or both of these sites destabilizes Fra-2, making it susceptible to proteosomal degradation. Conversely, phospho-mimetic mutation of S320 and T322 facilitate stabilization of the protein. Stabilization of Fra-2 contributes to the ensemble of gene expression, leading to skeletal muscle differentiation.

### Regulation and expression of Fra-2 in skeletal muscle differentiation

Fra-2 is expressed in cultured skeletal muscle cells and satellite cells, indicating that it may be important for skeletal muscle gene expression. ERK 1/2 signaling has also previously been implicated, as having a complex biphasic role in skeletal muscle differentiation.^27^ Here, we show that Fra-2 is a primary ERK substrate in response to cytokine signaling in myogenic cells. We previously reported CT-1 as an inhibitor of differentiation, and have implicated this cytokine as maintaining the undifferentiated state in MBs.^11^ CT-1 is an activator of the ERK 1/2 pathway and we now report that it targets Fra-2, indicating the possibility that AP-1 might be involved in maintenance of the undifferentiated state in myogenic cells. AP-1 expression is maintained in the ‘reserve population' in a differentiated C2C12 culture. These reserve cells are stochastically determined as cells that remain in a quiescent undifferentiated state, despite the surrounding clonally equivalent cells forming differentiated MTs.^28^ In addition, our observations that Fra-2 is expressed in Pax7-positive satellite cells and not in the differentiated myonuclei support a possible role in adult muscle progenitor cells. These preliminary data imply a previously unexplored role for AP-1 in regulating the reserve population (mononucleated cells in differentiation conditions) in an undifferentiated state and also satellite cells in mature muscle. Consistent with our data, AP-1 may be downregulated in MT, as multinucleated cells become unresponsive to ERK 1/2 signaling. Also, it is possible that secreted factors from MT impede differentiation of adjacent MBs, which establishes the reserve population by activating a paracrine signal pathway.^29^ If the cells become differentially responsive to ERK 1/2 signaling, then Fra-2 would be unphosphorylated at S320 and T322 in differentiating MTs when ERK signaling is downregulated, which could be a mechanism to destabilize Fra-2 protein and promote its proteosomal degradation. Our observation that Fra-2 levels are reduced in differentiating cells, and maintained in quiescent undifferentiated cells and satellite cells is consistent with this idea.

### S320-mediated Fra-2 protein stability is dependent on T322 phosphorylation

Interestingly, other Fos family members such as c-Fos and Fra-1 have a conserved serine at the site corresponding to S320 of Fra-2 in their C terminus.^17^ Moreover, the site corresponding to T322 of Fra-2 is also conserved on c-Fos and Fra-1, but has not been reported to influence the stability of these proteins. Here, we show that T322 of Fra-2 is vital for efficient phosphorylation and stabilization of Fra-2 primarily through its effect on S320 phosphorylation. Our data suggest that this site may function as a priming site for S320 phosphorylation; however, more detailed structural studies are required to further investigate the role of T322 (or its analogous residue in the other Fos proteins) in tandem with S320 for Fra-2 stabilization.

### AP-1 as a direct regulator of a variety of muscle-specific genes

Recently, Cao *et al.*^30^ reported that a large subset of genes regulated by MyoD in skeletal muscle cells are enriched with AP-1 sites. These data suggest that AP-1, similar to MyoD, may regulate a number of genes in the muscle lineage. Many of these genes prove to be downregulated during myogenesis. These differential effects of AP-1 at different gene loci reflects a common theme of modern transcription factor biology in which the effect of a particular factor on a target gene is dependent on the combinatorial influence of cofactors and other transcription factors at any given promoter/enhancer, as well as the myriad post translational events that converge on each transcriptosome. There are still a number of important paradoxes to explain with regard to AP-1 and its role in a variety of cellular contexts and processes. Part of the answer may lie in the promiscuous nature of AP-1 in its ability to interact with a diverse network of factors that may lead to complex transcriptional outcomes that are dependent on higher order transcriptosome network dynamics.

### Fra-2 as part of the AP-1 complex

The variation in AP-1 complex composition can dictate how it will function.^12^ Fra-2 is one component of a functional AP-1 complex that can also comprise of Jun-Jun, Jun-Fos or Jun-ATF2 dimers.^31^ In differentiating skeletal muscle, our data demonstrate that Fra-2 is the primary AP-1 subunit-binding DNA that dimerizes with either c-Jun or JunD.^12^ This data further suggest that Fra-2 is the major regulator in AP-1 complexes formed in myogenic cells. Thus, establishing a role for Fra-2-c-Jun and Fra-2-JunD complexes could provide insight into differential gene regulation by AP-1 complexes. In other systems, it has been determined that AP-1 function differs depending on its dimer composition.^32^ Differential subunit recruitment of cofactors is also a factor that might influence AP-1 specificity. For example, Trip6, a LIM domain protein, was found to interact with Fos family members but not Jun proteins,^33^ which illustrates an additional level of regulation for Fos proteins, possibly including Fra-2.

### Functional specificity and redundancy for AP-1 components

In an attempt to characterize the role of individual AP-1 subunits, some groups have generated and analyzed AP-1 knockout mice. Deletion of *c-jun*, *junB* or *fra-1* are found to be embryonic lethal, whereas mice lacking *jund*, *c-fos* or *fra-2* have specific organ defects.^34^ To date, no skeletal muscle defects have been reported for any of the AP-1 subunits. Interestingly, the *fra-*2 homozygous null mouse dies within 1 week of birth and is runted, thus implicating a possible lack of postnatal muscle growth based on the fact that skeletal muscle constitutes the heaviest contributor to body mass. Conditional knockouts have provided more information on the role of individual AP-1 proteins in specific tissue types. For example, c-Jun, when deleted in hepatocytes, impairs liver regeneration. Interestingly, some studies have analyzed the possible redundancy of AP-1 protein function by ‘knockin' strategies. For example, JunB was knocked into the c-Jun locus and was found to largely rescue the embryonic lethality of c-Jun homozygous deletion.^35^ Also, Fra-1 was knocked into c-Fos-deficient mice and found to rescue c-Fos knockout defects.^36^ Contrary to the gene targeting data that suggest specific functions of different AP-1 components in different tissue or cell types, the ‘knockin' data suggest that, to some degree, the specificity of AP-1 subunits may be more closely related to their spatial and temporal pattern of expression than differences in the properties of the individual subunits. This is indeed a controversial idea in view of the many studies that have shown unique properties of distinct AP-1 components, necessitating further clarification.

In summary, we have observed restricted expression of Fra-2 in the reserve and satellite cell populations in skeletal muscle. siRNA-mediated reduction of Fra-2 increases the commitment of cells to the differentiation program, suggesting a possible role for Fra-2 in satellite cells. In addition, we have identified and characterized two phosphorylation sites on Fra-2 that are targeted by ERK 1/2 signaling that potently regulate Fra-2 stability in skeletal muscle. Fra-2 expression is restricted to cells that are maintained in the undifferentiated state, suggesting that the signal-dependent stabilization of Fra-2 and its contribution to the AP-1 complex may be an important contributor to satellite cell function in response to a variety of cytokines.

## Materials and Methods

### Cell culture

The C2C12 cell line was purchased from American Tissue Culture Collection (ATCC), Cedarlane (Burlington, ON, Canada). Cells were maintained in growth media consisting of 10% fetal calf serum in Dulbecco-modified Eagle's medium (DMEM; Gibco, Burlington, ON, Canada) supplemented with 2 mM L-glutamine (Invitrogen, Burlington, ON, Canada) and 100 *μ*g/ml penicillin/streptomycin (Invitrogen). Cells were induced to differentiate at 80% confluency using differentiation media^6^ consisting of 2% horse serum in DMEM supplemented with 2 mM L-glutamine (Invitrogen) and 100 *μ*g/ml penicillin/streptomycin (Invitrogen).

### Fractionation of MTs and reserve cells

C2C12 cells were allowed to differentiate for 96–144 h in DM. Media was removed from the plate and cells were washed twice with cold 1 × PBS (phosphate-buffered saline) followed by addition of 1 ml of 0.125% trypsin diluted in 1 × PBS. Cells were inspected using an Axiovert 25 (Carl Zeiss, Toronto, ON, Canada) light microscope for MT contraction. On visual observation of MT contracture, trypsin was removed and 1 ml of cold 1 × PBS was added. The plate was gently swirled to dislodge MT, which were then collected in a 1.5 ml tube. The plate was further washed with cold 1 × PBS to remove residual MT after which the reserve cells (which remained on the plate) were scraped off into a 1.5 ml tube.

### Cloning and mutagenesis

The Fra-2 ORF was cloned into the pGEX-4T vector, pcDNA3 (Invitrogen) or in frame downstream of an EGFP tag. Site-directed mutagenesis was carried out using the QuikChange Multi site-directed mutagenesis kit (Stratagene, Agilent technologies, Mississauga, ON, Canada) for construction of the Fra-2 phospho-mutations, following manufacturer's protocol.

### Antibodies and other reagents

The following antibodies were purchased from Santa Cruz, Dallas, TX, USA: Fra-2 Q-20 (sc-604), Fra-2 L-15 (sc-171), c-Jun (H-79) (sc-1694), actin (I-19) (sc-1616-R), dsRed (C-20) (sc-33354), MyoD (M-318) (sc-760), ERK1 (C16) (sc-93), donkey anti-goat IgG-HRP (sc-2020). The following antibodies were obtained from Cell Signalling Technology, New England Biolabs Ltd, Whitby, ON, Canada: MEK 1/2 (no. 9122), phospho-MEK 1/2 (no. 9121), phospho-p44/p42 MAPK (Thr202/Tyr204) (no. 9106), phospho-Fra-1 (no. 3880), phospho-c-Jun (no. 9261). Myogenin (clone F5D) and MyHC (clone MF20) monoclonal antibodies were derived from hybridomas provided by the Developmental Studies Hybridoma Bank. Goat anti-rabbit IgG-HRP (170-6515) and goat anti-mouse IgG-HRP (170-6516) were from Bio-Rad Laboratories, Mississauga, ON, Canada. Lyophilized CT-1 (438-CT) was obtained from R&D Systems, Cedarlane, Burlington, ON, Canada. PD 98059 (no. 9900) was purchased from Cell Signalling Technology. MG132 (C2211) was purchased from Sigma-Aldrich, Oakville, ON, Canada.

### Transfection

Cells were seeded at a density of 12.5 × 10^3^ cells/well for six-well plates and 1.0 × 10^5^ cells/plate for 100 mm plates. Transfection were carried out using standard HEPES-buffered saline–CaCl_2_ phosphate-mediated transfection method using a total of 5 *μ*g of DNA for six-well plates and 25 *μ*g for 100 mm plates. Expression vectors used included pcDNA3_Fra-2, pcDNA3_Fra-2 DEF and dsRed2, and Fra-2 S120A, S230A, S320A, Fra-2 S120A, S200A, S230A, Fra-2 S120A, S200A, S230A, S320A, Fra-2 S320A, Fra-2 T322A, Fra-2 S320A T322A, Fra-2 S320D and Fra-2 T322D. Luciferase reporter constructs include pGL4-10_luc, pGL4-10_*myogenin*_luc and pRL_Renilla (Promega, Madison, WI, USA). Three siRNAs (Mission siRNA ID's: SASI_Mm01_00201000, SASI_Mm01_00201002, SASI_Mm01_00201004) targeting mouse Fra-2 were obtained from Sigma-Aldrich. They were reconstituted in nuclease-free water (Ambion, Burlington, ON, Canada), and 15nM of siRNA was transfected into cells using lipofectamin (Invitrogen) in serum-free media.

### Protein extraction

For total cell lysate analysis, media was aspirated from plates and cells were washed twice with cold 1 × PBS and scraped into 1.5 ml tubes. Cells were pelleted at 1.5 × 1000 × *g* and resuspended in Nonietp-40 (NP-40) lysis buffer (50 mM Tris, 150 mM NaCl, 0.5% NP-40, 2 mM EDTA, 100 mM NaF and 10 mM Na pyrophosphate) supplemented with protease inhibitor cocktail (Sigma-Aldrich, Oakville, ON, Canada, P8340), 1 mM NaV (Bioshop, Burlington, ON, Canada) and 1 mM PMSF (Sigma-Aldrich). Cytosolic and nuclear extraction was performed using a NE-PER nuclear protein extraction kit (Thermo Scientific, Lafayette, CO, USA, no. 78833) Protein concentrations were determined using the Bradford protein assay (Bio-Rad).

### Luciferase reporter gene assays

Media was aspirated from cells grown in six-well dishes and cells were washed twice with cold 1 × PBS. Cells were scraped in luciferase lysis buffer (20 mM Tris pH 7.4 0.1% Triton X-100) and lysate was transferred to 1.5 ml tubes. Samples were briefly vortexed and spun at maximum speed for 15 min at 4 °C. Samples were aliquoted into tubes, and luciferase assay substrate (E1501) or renilla assay substrate (E2820), purchased from Promega, was added, and luciferase and renilla enzymatic activity was measured on a luminometer.

### IP and CIP treatment

Immunoprecipitation was carried out using the Exacta Cruz F kit (Santa Cruz, sc-45043) according to manufacturer's protocol. Briefly, 40 *μ*l of IP matrix was incubated overnight with 5 *μ*g of Fra-2 primary antibody. The IP matrix–antibody complex was washed three times with cold 1 × PBS and incubated overnight with 120 *μ*g of total cell lysate. IP matrix–antibody–protein complex was washed three times with cold 1 × PBS supplemented with protease inhibitor cocktail (Sigma-Aldrich, P8340), 1 mM NaV (Bioshop) and 1 mM PMSF (Sigma-Aldrich), before incubation with CIP (NEB, M0290S) or its buffer. A concentration of 2 × SDS sample buffer was added, and samples were boiled and loaded on a 10% SDS-polyacrylamide gel electrophoresis (PAGE) and analyzed by western blotting.

### Western blotting

A total of 20 *μ*g of total lysate was resolved on 10% (or 8%) SDS-PAGE and transferred onto Imobolion-P or Immobilon-FL PVDF membranes (Millipore, Fisher Scientific (distributor) Ottawa, ON, Canada). Membranes were blocked using 5% milk in 1 × PBS or 1 × Tris-buffered saline (TBS) containing 0.05% Tween-20 (TBS-T), and incubated with primary antibodies overnight at 4 °C. Membranes were washed with 1 × PBS or 1 × TBS-T and incubated with secondary antibody then washed with 1 × PBS or 1 × TBS-T. Immunoreactive bands were detected using ECL Chemiluminescence reagent (GE Healthcare, Mississauga, ON, Canada).

### GST purification

Human full-length ORF of Fra-2 was cloned in frame into a pGEX-4T-1 vector, which contains GST to generate a GST-Fra-2 fusion protein. GST-Fra-2 was transformed in BL-21-competent *Escherichia coli* cells. Isopropyl *β*-D-1-thiogalactopyranoside (IPTG; Bioshop, IPT001) was used to induce expression of the fusion protein. Cells were harvested and the fusion protein was purified via affinity binding on GST beads (Sigma-Aldrich, G4510).

### *In vitro* kinase assay

Bacterially expressed-purified GST (2 *μ*g), GST-Fra-2 (2 *μ*g) or MBP (NEB, P6021S; 2 *μ*g) were incubated with radioactive γ^32^P-ATP (Amersham, GE Healthcare) with or without purified ERK 2 (20 ng) kinase (NEB, P6080S). Samples were incubated at 30 °C for 40 min before addition of 4 × SDS sample buffer and boiled for 5 min. Samples were resolved on an 8% SDS-PAGE, which was Coomassie stained to visualize bands. The gel was dried and exposed to film to capture light emitted by γ^32^P-ATP-labeled proteins.

### In-gel digestion and mass spectrometry analysis

The kinase assay as indicated above was repeated, replacing the radiolabelled γ^32^P-ATP with unlabeled ATP (NEB, P0756S), and the band shift was observed once again in a Coomassie blue-stained gel when GST-Fra-2 was incubated with ERK 2; however, an 8% SDS-PAGE was run to achieve greater resolution of the bands. The lower mobility-phosphorylated GST-Fra-2 band was excised along with the unphosphorylated GST-Fra-2 band. These bands were digested with trypsin overnight at 37 °C. Tryptic peptides were loaded onto a HPLC Chip (160 nl high-capacity sample enrichment column and 75 *μ*m × 150 mm SB-C18 separation column; Agilent Technologies, Santa Clara, CA, USA) and separated by flow rate at 300 nl per minute, with solvent A (0.2% (v/v) formic acid in water) and solvent B (100% acetonitrile) and the following gradients: at 0, 50, 54 and 56 min after injection with 3, 35, 80 and 100% solvent B, respectively. The LC-MS/MS analysis was carried out using an Agilent 1100 HPLC chip and 6340 ion trap system with MS scan range from 300 to 1300 m/z and back-to-back CID/ETD (collision-induced dissociation/electron transfer dissociation). Thirty seconds dynamic exclusion was applied to the precursor, previously selected for MS/MS twice. Raw data files from LC-MS/MS were searched against a custom protein sequence, representing GST-Fra-2 using Spectrum Mill MS Proteomics Workbench (v03.03.084, Agilent Technologies, Canada). The Data Extractor utility program detected peaks, assigned precursor charges where possible (for those not successfully determined, 2+ to 5+ were considered), filtered MS/MS spectra by quality (spectra with peak number >4 and sequence tag length >2 were kept for MS/MS search), centroided the MS/MS spectra, merged nearby MS/MS spectra from the same precursor by default MS/MS similarity criteria and generated peak lists. Peak lists were searched by the following criteria: two missed trypsin cleavages, fixed modification (carbamidomethylation on cysteine), variable modifications (oxidized methionine, pyro-glutamic acid modification at N-terminal glutamines, phosphorylated-serine, -threonine, and -tyrosine), precursor mass tolerance +/− 2.5 Da and product mass tolerance +/− 0.7 Da. The spectra identified by Spectrum Mill to be phopshorylated were manually verified and reported.

### Immunocytochemistry

Cells were grown to the desired state and media was removed. Cells were washed with 1 × PBS and fixed with 70% methanol. Cells were blocked in 5% milk in 1 × PBS and incubated with MyHC primary antibody. Primary antibody was removed and cells were washed with 1 × PBS and incubated with anti-mouse secondary antibody HRP conjugated. A final concentration of 10mg/ml of DAB (Sigma-Aldrich, D8001) and 3% hydrogen peroxide (Sigma-Aldrich, 216763) were used as a substrate and the nuclei were stained with hematoxylin (Sigma-Aldrich, H3136).

### Limited proteolytic cleavage

*In vitro* kinase was performed as described above and the reaction products were incubated with trypsin (Roche, Laval, QC, Canada) at 16 °C for the indicated times. At each time point, 4 × SDS loading buffer was added to stop the cleavage. Samples were boiled and analyzed by western blotting.

### Primary skeletal muscle fiber isolation and immunoflourescence analysis

Myofibers were isolated from the muscle of 4-extensor digitorum longus (EDL)-month-old female C57Bl/6 mice as described in detail by Otto *et al.*^37^ (2008). Briefly, undamaged EDL muscles were dissected with both tendons intact and the single fibers liberated through digestion with 0.1% type I collagenase in DMEM at 37 °C 5% CO_2_. Heat flame-tapered glass pipettes were used to plate isolated single fibers into floating culture wells containing DMEM supplemented with 10% horse serum and 0.5% chick embryo extract for up to 72 h. For immunocytochemistry, myofibers were fixed in 2% paraformaldehyde in PBS for 10 min and washed three times in PBS. Myofibers were permeabilized in a solution of 20 mM HEPES, 300 mM sucrose, 50 mM NaCl, 3 mM MgCl_2_ and 0.5% Triton X-100 (pH 7) at 4 °C for 15 min and incubated in blocking wash buffer (5% newborn calf serum in PBS containing 0.01% Triton X-100) for 30 min before antibody incubation. Antibodies were diluted and pre-blocked in wash buffer for 30 min before addition to the myofibers. Primary antibodies used were: monoclonal mouse anti-Pax7 and anti-myogenin (clone F5D, both from Developmental Studies Hybridoma Bank) 1 : 1, Fra-2 (Santa Cruz Q-20) 1 : 2000 and phospho-Fra-1 (Cell Signalling Technology, no. 3880) 1 : 1000. All primary antibodies were incubated with fibers overnight at 4 °C. Primary antibodies were visualized using the following secondary antibodies: Alexa Fluor goat anti-mouse 594 (A11032 Molecular Probes, Paisley, UK), Alexa Fluor goat anti-rabbit 488 (A11034 Molecular Probes). Secondary antibodies were used at 1 : 200 and incubated at room temperature for 45 min. All myofibers were mounted in Fluorescent mounting medium (DAKO cytomation, Cambridgeshire, UK) containing 7.5 mg/ml DAPI for nuclear visualization. Mounted myofibers were analyzed using a Zeiss Axioscope fluorescence microscope (Zeiss, Cambridge, UK), and images were captured using an Axiocam digital camera system (Zeiss) and Axiovision image analysis software (version 4.7, Zeiss).

## Abbreviations

AP-1: activator protein-1
CT-1: cardiotropin-1
EDL: extensor digitorum longus
ERK 1/2: extracellular signal-regulated kinase
MAPK: mitogen-activated protein kinase
MB: myoblast
MT: myotube
MyHC: myosin heavy chain
