# Supplementary figures and images for: Signal-dependent fra-2 regulation in skeletal muscle reserve and satellite cells
Source: Cell Death Dis. 2013 Jun 27;4(6):e692–. doi: 10.1038/cddis.2013.221 (PMC3702306; doi:10.1038/cddis.2013.221)

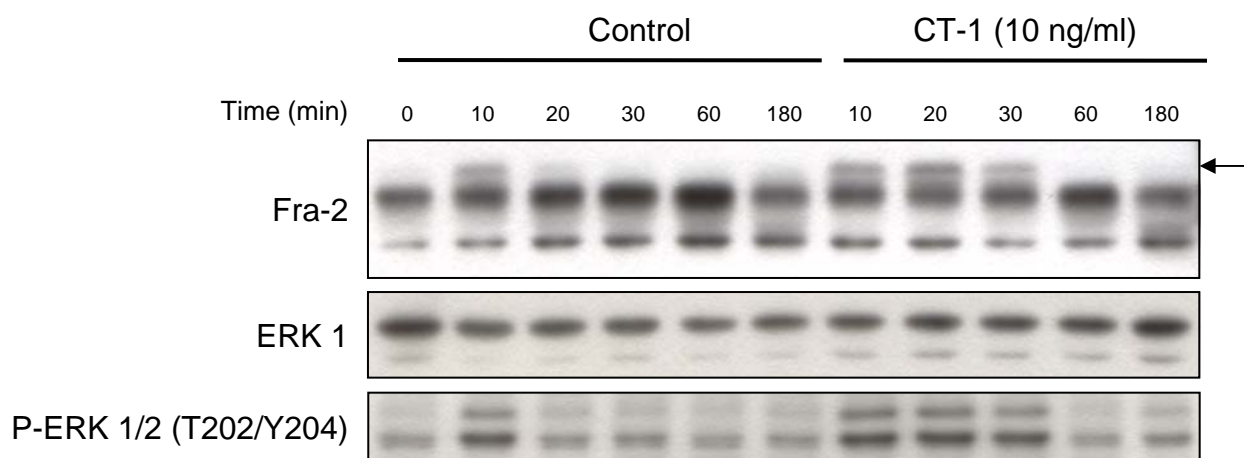

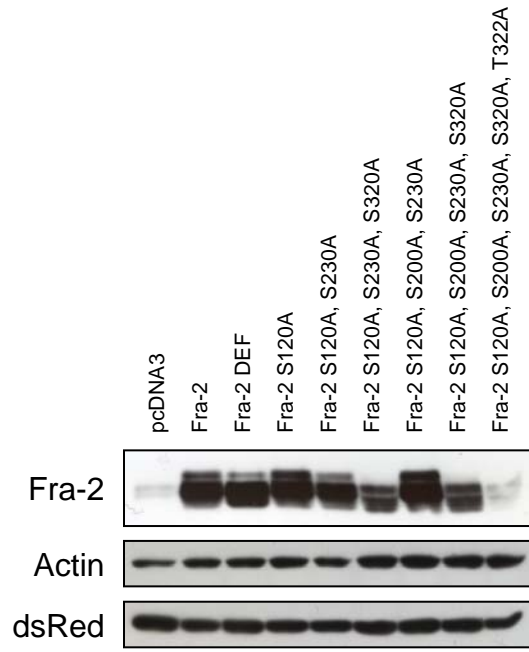

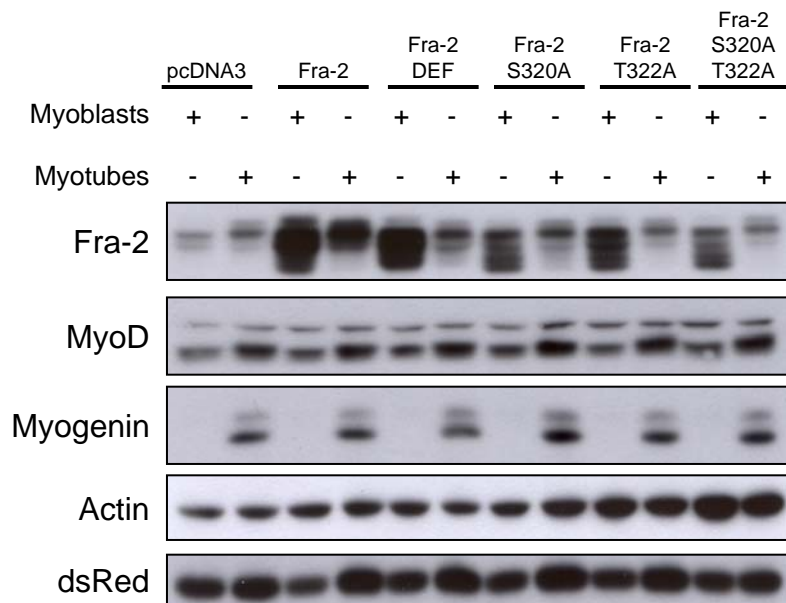

Supplement: Supplementary Figures [file cddis2013221x1.pdf]
