# Supplementary material for: Signal-dependent fra-2 regulation in skeletal muscle reserve and satellite cells
Source: Cell Death Dis. 2013 Jun 27;4(6):e692–. doi: 10.1038/cddis.2013.221 (PMC3702306; doi:10.1038/cddis.2013.221)
Supplement: Supplementary Figure Legends [file cddis2013221x2.pdf]

Figure legends for supplementary figures

S1. CT-1 transiently activates Fra-2 in C2C12 cells. C2C12 cells were serum starved for 8h followed by treatment with CT-1 (10ng/ml) and harvested at the indicated time points. Western blot analysis was performed to determine CT-1 effect on Fra-2 protein levels.

S2. ERK 1/2 targets S320 and T322 are regulated sites on Fra-2. Combinatorial mutation for Fra-2 phospho-sites were generated and transfected into C2C12 cells. Western blotting was used to determine expression of the mutated Fra-2 constructs. Actin was used as a loading control and dsRed as a marker for transfection efficiency.

S3. Neutralization of S320 and T322 on Fra-2 decreases protein expression. C2C12 cells were transfected with wild-type Fra-2, Fra-2 DEF, or neutralization mutation for S320 and T322. Expression of Fra-2 and its mutation were assessed by Western blotting in MB and MT. Actin was used as loading control and dsRed as a marker for transfection efficiency.
